# COVID-19 Pandemic Lockdown and Religious Mediatization of Social Sustainability. A Case Study of Romania

**DOI:** 10.3390/ijerph18052287

**Published:** 2021-02-25

**Authors:** Mihaela Alexandra Tudor, Anamaria Filimon Benea, Stefan Bratosin

**Affiliations:** 1 Philosophy and Communication Sciences Department, West University of Timisoara, 48901 Timisoara, Romania; anamaria.filimon@e-uvt.ro; 2 IARSIc-CORHIS EA7400, Université Paul Valéry Montpellier, 34090 Montpellier, France

**Keywords:** COVID-19 pandemic, health, mediatization, production of meaning, religious media institutions, Romanian religious denominations, social sustainability, social worlds, spirituality, well-being

## Abstract

This article presents an empirical study on the institutional audiovisual mediatization of social sustainability made by the eighteen religious denominations officially recognized in Romania during the period of the COVID-19 pandemic onset. Research is undertaken based on the mediatization theories. Specifically, it highlights and discusses the conditions for producing the meaning of social sustainability as a result of religious mediatization during the months of March, April and May 2020, a period with strong religious connotations since it involved the dates of the major annual feasts celebrated by the three majority monotheistic religions, i.e., the Christian Easter, the Muslim Ramadan and the Jewish Passover. As a result, we noticed that the production of meaning in terms of social sustainability was simultaneously anchored in the accumulation of four contextual “social worlds”: (a) that of social transformation induced by mediatization, (b) that of the COVID-19 pandemic, a crisis that is neither social, economic, or environmental, but with consequences on the three levels of reality mentioned above, (c) that of spirituality during the time of the great monotheistic religious feasts and (d) that of the national culture of Romania, statistically the most religious country of the European Union.

## 1. Introduction

The article presents the results of an empirical research on the mediatization of social sustainability in times of crisis. Specifically, it highlights and discusses the conditions for producing the meaning of social sustainability (we use the notion of “social sustainability” according to the definition given by the Oxford Institute for Sustainable Development (OISD): “*Social sustainability concerns how individuals, communities and societies live with each other and set out to achieve the objectives of de-velopment models, which they have chosen for themselves, taking also into account the physical boundaries of their places and planet earth as a whole. At a more operational level, social sustainability stems from actions in key thematic areas encompassing the social realm of individuals and societies, ranging from capacity building and skills development to environmental and spatial inequalities (…). In this sense, social sustainability blends traditional social policy areas and principles such as equity and health, with issues concerning participation, needs, social capital, the economy, the environment, and more recently, with the notions of happiness, well being and quality of life*.” [1]).

We distinguish between the “mediatization of religion” and “religious mediatization”. Unlike the “mediatization of religion”, which concerns only religion and is operated by all religious and secular media, “religious mediatization” refers to mediatization originating only from religious media. It includes both religion and all the symbolic forms (art, science, economics, politics, etc.), components of the public sphere.) as a result of religious mediatization during the months of March, April and May 2020 when the specific measures imposed by the COVID-19 pandemic were introduced. More precisely, the objective of this paper is to highlight—in terms of media production and information content—the way in which the media of religious organizations have communicated on issues relating to social sustainability during a health crisis. The field of observation in question is limited to the national territory and the legislative framework of Romania. The object of the study is the mediatized communication (the processes of production of meaning in a practiced context, cf. Author) of social sustainability. This process is understood as an intellectual construct shaped by the social transformation induced by mediatization, by the exceptional circumstances specific to the COVID-19 pandemic, by the observance dates of the religious holidays of the great monotheistic religions and by the religious predisposition of the national culture in Romania. Although the detailed description of this aspect is given elsewhere see [2,3], a brief discussion of the empirical context that has shaped our observations is essential. Statistically, Romania—which overthrew its communist regime in December 1989—is the most religious country of the European Union (EU) with 89.9% Orthodox Christians and 1% non-believers [3,4]. If at the institutional level of the EU, the border, which followed the limits of the national territory of the Romanian State, had to quickly submit to the technical requirements of adapting to the new configuration, the sociocultural symbolic borders are still the site of a very fruitful unfinished socio-political reorganization, a reorganization which draws on the traditional cultural background of Orthodox Christianity focusing on national identity [5,6] and the old programming ideology of the new power inclined to transmute communism into populism in favor of a political opportunism now perceived by the population as a process of democratization [2,7,8]. The emerging media and especially social media, with Facebook as a priority, are a prolific communicational embodiment of the return to borders, of the nationalism of Orthodox Christian origin and of post-communist populism.

In Romania, religion and politics together represent an apparatus to produce meaning and legitimate power in a democracy deeply built on a national citizenship and identity of the religious affiliation: Christian Orthodoxy. As Huntington said, Romanians see the advantage of being part of the West and being incorporated into its institutions, but they remain attached to their Orthodox tradition [9]. In this specific context, we develop an empirical research herein that draws on social sustainability model and media and communication studies theoretical framework [10,11,12,13,14,15,16].

## 2. Theoretical Background

Research on sustainability is abundant and covers not only very different, but also divergent approaches (sustainability is understood here as not a disciplinary field (such as sociology, philosophy, economics, management, etc.), but a trans- and multidisciplinary topic (such as minorities, violence, security, etc.). In this context, the role of this bibliographic framework becomes absolutely necessary to indicate to the reader the discipline in which the work on the subtopic of “social sustainability” (the general topic being “sustainability”) makes sense). The most eloquent expressions of this heterogeneity and multiplicity of these scholarly studies are (a) the impressive list of books that report the results of current research on sustainability, constantly enriched by new titles [17,18,19,20,21,22,23] and (b) the editorial production of scientific journals such as *Sustainability*, *Ecosystem Health and Sustainability*, *Current Opinion in Environmental Sustainability, Energy Sustainability and Society, Environment Development and Sustainability, International Journal of Agricultural Sustainability, International Journal of Sustainability in Higher Education, Nature Sustainability, Sustainability Accounting Management and Policy Journal, Sustainability Science,* etc. However, these studies agree on two points: (a) the tripartite structure of the concept of sustainability—the economy, the society, and the environment aspects and (b) the cultural conditioning of these three pillars of sustainability [24,25,26,27,28,29,30,31,32,33,34,35,36,37,38,39,40,41,42,43,44,45,46,47,48,49,50,51,52,53,54,55,56,57,58,59,60] that informs on the role of culture on the understanding and implementation of sustainable practices by considering seven pairs of indicators: (a) “Engagement and Identity”, (b) “Performance and Creativity”, (c) “Memory and Projection”, (d) “Belief and Meaning”, (e) “Gender and Generations”, (f) “Enquiry and Learning, (g) Health and Well-being”.

In this context, studies on the impact of beliefs and meanings of social sustainability have highlighted the role of religion in the design and communication of the objectives of the U.N. Sustainable Development Goals. Thus, several studies [61,62,63,64,65,66,67,68] underline the major role of religion in the communication on social sustainability. More precisely, this research highlights the close relationship between the role of religious beliefs and practices in persuading and engaging spiritual communities in favor of social sustainable behaviors and life. In this field, the tripartite links between religion, sustainability and the media environment have been, for several years, one of the major directions of research and scientific production in social sciences [69,70,71,72,73]. This article falls into the category of scholarly works. It attempts to produce empirical results, showing how the specific impact of religious media on the production of meaning of social sustainability can be multiplied and how the institutionalized religious media seized on the mediatization of social sustainability during the lockdown due to the epidemic crisis of the Covid-19.

More precisely, this article presents the results of an empirical research on the institutional audiovisual mediatization of social sustainability made by the eighteen religious denominations officially recognized in Romania. This research is undertaken based on the theory of mediatization.

Mediatization is an approach that corresponds to the new generation of communication and media theories [74,75]. It proposes a multitude of functional hypotheses on the basis of the explanations concerning the transformations of contemporary society: (a) the media are agents of change, (b) the phenomena and domains of reality submit to the logics of the media in terms of institutional regulation, symbolic content and individual practices, (c) the media acquire social and institutionalized functions and participate in the construction of the imagination of society and culture [74,75,76]. This approach relies on the thesis that social processes and phenomena cannot be studied and understood outside of the media and not all media can be understood outside of society.

The notion of mediatization appears in the Anglo-Saxon scientific literature with the contributions of Knut Lundby [77], Andreas Hepp [78] and Stig Hjarvard [79,80]. The field of communication studies adopts the concept as theoretical and empirical studies gain importance. Two main directions are at the base of this approach, i.e., the “institutionalist” and the “social constructivist” [81,82,83,84]. According to the institutionalist approach [79,85,86,87], mediatization is an adaptation of the different systems and domains (social, political, religious, etc.) to media logics. According to the social constructivist approach, mediatization is a process in which the evolution of information and communication technologies leads to transformations in the communicative construction of culture and society [82]. In the wake of these directions, other schools emerge: in Latin America, the Brazilian school focused on the study of the mediatization of religion, in Europe, France, a network research focused on anthropological mediality as variable of mediatization [88,89]. Our article focuses on mediatization from the combined theoretical perspective of the two traditions, since the objective of research on mediatization is ultimately the same: to critically analyze (critical analysis is understood here in Foucauldian terms, as “a matter of saying that things are not right as they are. It is a matter of pointing out on what kind of assumptions, what kinds of familiar, unchallenged, unconsidered modes of thought the practices that we accept rest… Criticism is a matter of flushing out that thought and trying to change it: to show that things are not as self-evident as one believed, to see that what is accepted as self-evident will no longer be accepted as such. Practicing criticism is a matter of making facile gestures difficult.” [90]) the relationships between transformations in the media, on the one hand, and communication and the transformations of culture and society, on the other hand [75,76,91]. The notion of context is essential [92].

From the point of view of the mediatization approach, we understand social sustainability as a mediatized world [82]. More precisely, it is a materialization of the mediatization, a reality breakdown, a social world. In this context, we are mainly interested in understanding how the meaning of social sustainability is established through the mediated and mediatized forms of interaction which are the religious media.

The relevance of the religious element here is justified by (a) the close, multidimensional relationship between the universe of symbolic meanings of religion and the applied concept of sustainability and (b) the articulation in the public sphere of media logics and the logics of religious logics. More precisely, religion and media are an interdependent phenomena since religions engage the media and the media are engaged by religions [93,94,95,96,97]. Indeed, since the late 1980s, several research publications have already highlighted the deep link between religion and sustainability [98,99,100,101,102,103,104]. In these studies, religion and sustainability are treated as two complementary realities: *“(...) the key motivation for participating in the quest for a better, more sustainable world was often religious (…). (…) the emergence of sustainability and the resurgence of religion may be viewed as complementary trends*” ([103], p. 70). Moreover, it is on this basis that the major principles of “*sustainability research seeking to advance knowledge on the relationship between religion and social values*” are laid ([66], p. 1355). On the other hand, studies on the role of religious actors in the public sphere, directly or implicitly, highlight the media formatting of religious communication on issues of sustainability. Thus, religious communication under the influence of media logics appears in the public sphere as (a) crisis communication, debate and campaigning to raise awareness of sustainability in the public sphere [105,106,107,108,109,110,111,112,113], (b) participation in projects related to sustainability [114,115] and (c) diffusion of values and world views that support environmentally aware attitudes and actions sustainability [99,100,116,117,118,119,120]. Research that falls within this epistemological-methodological triangle—sustainability, media, religion—highlight numerous interactive reciprocities between religion and media. These interactive reciprocities are expressed in different forms: (a) the contribution of the media to the construction of the *religious* (magazines, religious web site, mediatization of religious events…), (b) the presence of organized religion into the media (churches, the mediatization of religious events related to the religious institution…), and (c) the presence of religious elements in the media [96,97,121,122,123,124].

Based on this, the article formulates research question and observation features, articulates methods, and presents the results.

## 3. Methodology

The objective of the research is to observe the treatment of social sustainability by the institutional religious media of the eighteen formally recognized religious denominations in Romania during the period of the COVID-19 pandemic onset. We carried out a media monitoring throughout the months of March, April and May 2020, a period with a strong religious connotation since it involved the dates of the major annual holidays celebrated by the three majority monotheistic religions, i.e., the Christian Easter, the Muslim Ramadan and the Jewish Passover. The recognized religious denominations in Romania (Table 1) cover 99.63% of the country’s population. The recognized denominations are all expressions of one of the three great monotheistic religions of the world. Without being formally banned, other religions have no official state recognition. The distribution of believers between the three major monotheistic religions is very disproportionate in favor of Christianity which brings together 99.64% of declared believers, while Islam only 0.34% and Judaism 0.02%. Of the eighteen recognized denominations, 16 are Christian; out of the 16 Christian denominations, three are Orthodox, three Catholic, four Protestant and six neo-Protestant or assimilated to neo Protestantism. This categorical designation also contains very unequal membership in the number of faithful. Out of all the Christian faithful in Romania, the Orthodox denominations have 86.70% of believers, Catholics 5.42%, Protestants 3.41% and neo Protestants and assimilated to neo Protestantism 4.47% [125] This sociological reality is an important methodological landmark in the economy of the present study, because it makes possible to give meaning to a media reality which by its “media” nature (a) transgresses physical, institutional, ideological, doctrinal, etc. boundaries and (b) blurs—“make liquid” [126]—quantitative determinations which are objectively attached to recognized religious denominations in Romania. This aspect will be dealt within the “Results” and “Discussions” sections.

The religious media landscape in Romania was already presented in a previous study which revealed important information about (a) main religious printed media, broadcast media, Internet and social media, and new agencies; (b) financing; (c) relations between the religious media and the state, and positioning in relation to the state policies; (d) relations between the religious media and the public service media; (e) interfaith relations; and (f) religious media development trends [91]. All useful details concerning denominations with their own media—i.e., religious print media and news agencies, television and radio, the engagement of the religious media with the new media and Internet—could be consulted through this study. We emphasize here that the Orthodox Christian Church and the Seventh-Day Adventist Church are the most present denominations in the Romanian public sphere through their own institutional media (old and new) [91].

The data collected, including a substantial corpus of discursive material, was then analyzed with the methodological tool of content analysis. We made this choice for several reasons: (a) the object of content analysis is communication [127,128], (b) the content analysis takes into account the dynamics of social representations and (c) the content analysis takes into account the “production/reception” of the content of the media message as a symbolic universe of meaning production, as an ecological universe (context) and as a form of social interaction [129]. Content analysis is an instrument that helps to identify the meaning of the statements/formulations, on the one hand, and, on the other, to establish their relevance for the receiver, that is to say the relevant meanings to the audiences.

More specifically, we undertook: (a) the identification of social sustainability indicators constituting the discursive universe of media messages by identifying the categorizations (*thematic content analysis*) (b) the identification of the presence or absence of an indicator in media messages and the interpretation of the meaning of the message associated with the indicators (*evaluative content analysis*) (c) the identification of the repetitions of the associations with indicators (*stereotyping analysis*), and (d) the identification of the popularity of indicators (frequency analysis).

We used as indicators the social sustainability factors [76] which synthesize the key themes of social sustainability [49,54,130,131,132,133,134,135,136]. More precisely, we observed the indicators corresponding to the two conceptual levels of social sustainability—physical well-being and quality of life/equity—as well as the key elements of social sustainability corresponding to the quality of life/equity (cf. Table 2).

Six observation features are at the basis of the methodological design:

(1) The denominational otherness of the selected media production.
(2) The quantitative gap between the indicators of social sustainability in the selected media production.
(3) The media balance between the two conceptual levels of social sustainability—physical well-being and quality of life/equity.
(4) The denominational disparity in the treatment of the two conceptual levels of social sustainability—physical well-being and quality of life/equity.
(5) The denominational specificity in the treatment of social sustainability.
(6) The accentuated and concordant intensity of the mediatization of certain indicators of social sustainability.

The selected corpus is composed of 99 audiovisual products disseminated by institutionalized religious media (i.e., TV and Radio broadcasts, videos on institutional websites, —and on social media—Facebook institutional accounts, press articles and press releases). The leading principle for selection was the surveying the themes related to the social sustainability indicators. This guiding principle has been applied indiscriminately to all types of productions and media (concerning the issues of the types of media productions and the comparison between media which are linked to sociological analyses, they are known and recognized for their trapping character. To avoid this pitfall, we have chosen not to enter into this logic and to consider the media production studied as a field of heterogeneous communicative action (a habermasian perspective). The content of each of these products has the discriminatory characteristic (we define the “discriminatory characteristic” as a characteristic which indicates a distinction. Concretely, in the constitution of our corpus, we did not retain the products which communicated only on one or two of these criteria. “Discriminatory characteristic” is not used in the social sense) of communicating by establishing links between (a) global news, that is to say the COVID-19 pandemic, (b) promotion of social sustainability and (c) religious issues. The following results are obtained within this methodological framework and limited to religious media only, without crossing over into the field of religion as theology, doctrine, dogma, ideology, etc. Therefore, we fail to treat religion in a doctrinal, theological, dogmatic, ideological sense or as an independent variable simply because our focus was on religious media of the main Romanian denominations. Mediatically, this concern was only marginally addressed. Instead, religion is used as an explanatory variable, because religious media appeal to religion as a strategy to maintain their institutional relevance.

## 4. Results

The analysis of the corpus showed that of the 18 officially recognized denominations in Romania, only 14 produced media content corresponding to the discriminatory characteristic retained in the methodology of this study. This explains the absence of the CCG, ELC, REC and SOD in the presentation of results. In what follows, we will present the results according to the working features retained in the methodological protocol of our research.

### 4.1. The Denominational Otherness of the Selected Media Production

**Figure 1:**
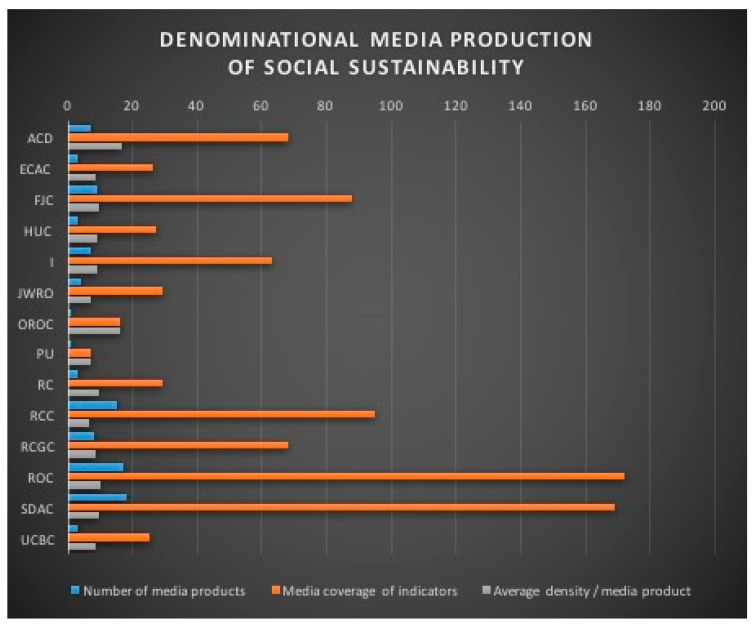
Denominational media production of social sustainability.

The religious mediatization of the issues related to social sustainability as a cultural phenomenon (i.e., collective, symbolic, material, experiential and sensitive phenomenon, as defined by [95,138] is the result of an unequal denominational contribution that is not directly related to the number of the faithful (for more details on statistics see INS [92,139,140]). For example, ROC and SDAC have substantially similar contributions, although Christian Orthodoxy is the majority denomination in Romania with 86.45% of the population, while Seventh Day Adventism is a religious minority with only 0.3% of the population. Thus the number of media productions varies from 18 for SDAC to 1 for OROC and PU (Figure 1). The coverage in terms of number of indicators is also irregular and is not necessarily related to the number of media production created by each denomination. For example, ACD and I, with seven media productions each, have a coverage of 68 and 63 indicators, respectively. Similarly, with 17 media productions, ROC has a coverage of 172 indicators, whereas SDAC, with 18 media productions, has a coverage of only 168 indicators. Finally, the average density of indicators per media productions appears to be different in terms of denomination, but without direct relation to the number of media productions or to the number of believers. OROC, for example, with a single media production, has an average density of 16 indicators/products, while SDAC, with 18 media productions, has an average density of only 9.38 indicators/productions (Table 3). The reasons for these heterogeneous differences are undoubtedly to be found in the institutional capacity of each denomination to adapt its theological discourse and to update it in relation to social, political and economic realities. The cases of ROC, which falls within a national organizational tradition and of SDAC, which falls under a global organizational logic, suggest that the organizational culture and the denominational doctrine of the faith are not potential obstacles to media activism in favor of the understanding and promoting social sustainability. Quite the opposite, these heterogeneous gaps show that the institutional function and its public image, as social challenges of religion, seem to play a major role among the agents of meaning in the religious mediatization of social sustainability. This is the case of ROC and SDAC, but also of ACD, FJC, I, RCC or RCGC, whose religious presence in the public sphere is a constant historical, pragmatic or political setting.

### 4.2. The Quantitative Gap between the Indicators of Social Sustainability in the Selected Media Production

**Figure 2:**
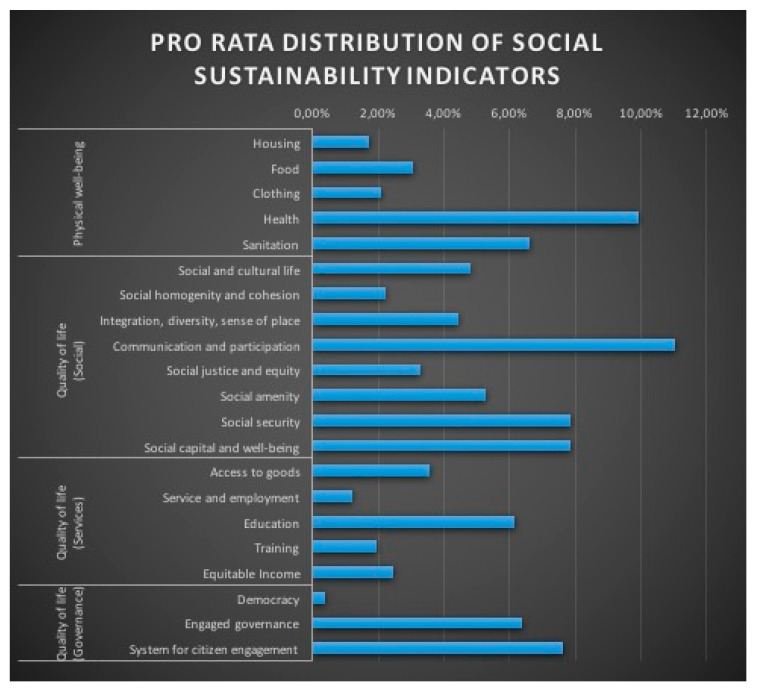
Pro rata distribution of social sustainability indicators.

The religious mediatization of the issues related to social sustainability is the result of a very irregular distribution of indicators in the total production of the corpus (Figure 2). Thus, on the whole of the religious media production, the indicator “Democracy” enjoys only 0.36% of the total distribution of indicators, while the indicator “Communication and participation” enjoys 11.05% of this distribution. The only exceptions are the “Social security” and “Social capital and well-being” indicators which have the same distribution, i.e., 7.86% (Table 4). These results show that the religious media treatment of the social issues related to sustainability is sectoral and unrelated to conceptual intercausality. The political measures taken because of the pandemic, for example, do not clearly give rise to questioning about “democracy” in the media, although the indicator “Communication and participation” appears to be the main indicator in the media, even ahead of the indicator “Health”,- strongly reinforced, one might suppose, by the health crisis.

### 4.3. The Media Balance between the Two Conceptual Levels of Social Sustainability—Physical Well-Being and Quality of Life/Equity

**Figure 3:**
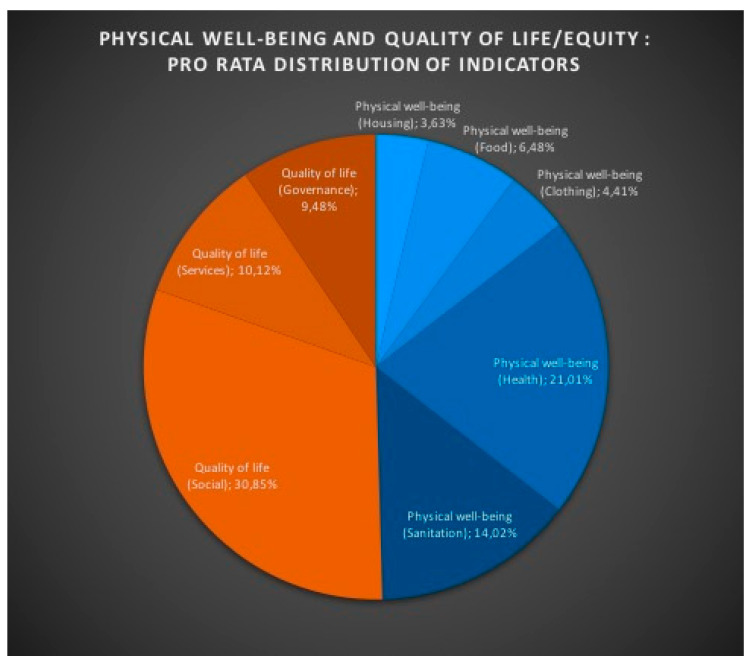
Physical well-being and quality of life/equity: pro rata distribution of indicators.

The religious mediatization of the issues related to social sustainability, despite the disparities in the media productions highlighted above, is based on a balance in the distribution of indicators corresponding to two conceptual levels: “Physical well-being” and “Quality of life” (Figure 3). However, this balance hides sensitive thematic unevenness (it is not a question of expressing here a possible expectation of equality, but quite simply of noting an empirical finding resulting from the measurements carried out, under identical conditions, on equivalent conceptual indicators; this effectively allows a logical and objective association in an analysis) within each level. Thus “Health” represents in “Physical Well-being” more than 42% of the total number of indicators, while “Housing” represents less than 7.5% of this level. The same observation can be made with regard to “Quality of life” where more than 61% of the indicators at this level relate to “Social” and only a little less than 19% relate to “Governance”. (Table 5) In view of these results, the media treatment of social sustainability by institutional religious media in Romania confirms that religion is not intended to distort the conceptual content of social sustainability. This religious media treatment is limited to producing meaning only inside this content, by confronting the variations of the denominational civic faith with the promises of the citizen science part of the concept of social sustainability.

### 4.4. The Denominational Disparity in the Treatment of the Two Conceptual Levels of Social Sustainability—Physical Well-Being and Quality of Life/Equity

**Figure 4:**
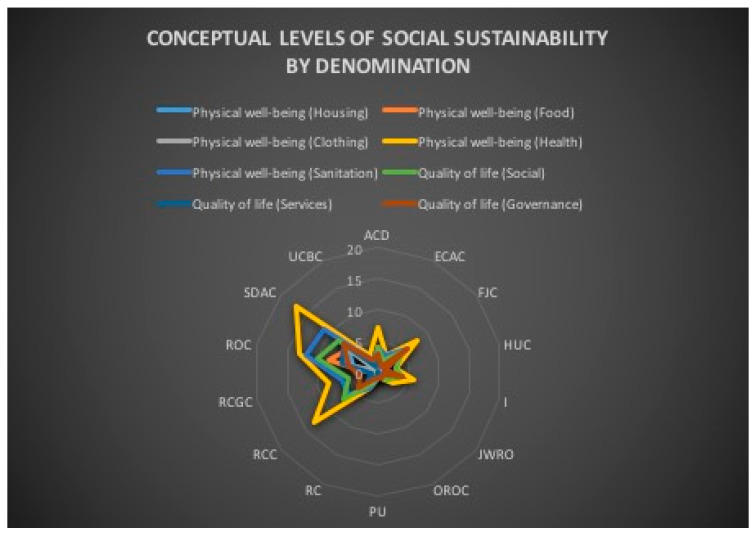
Conceptual levels of social sustainability by religious denomination.

The religious mediatization of the issues related to social sustainability, even if it is almost similarly represented in the distribution of indicators corresponding to the two conceptual levels, “Physical well-being” and “Quality of life”, when we consider all the denominations, appears less balanced if we consider the distribution of indicators corresponding to the two conceptual levels for each denomination separately (Figure 4). For example, with regard to “Physical well-being”, JWRO, OROC, PU and RCC do not have any contribution regarding “Housing”, “Food” and “Clothing”. In fact, the balance is essentially found overall by the contributions of ACD, ROC and SDAC. The same situation can be observed for the “Quality of life”. The lack of involvement of OROC, PU and UCBC for the “Social” and for the “Services” is filled by the media performances of ROC and SDAC. (Table 6). From this perspective, the trend is contrary to the previous observation on the conceptual levels of social sustainability—physical well-being and quality of life/equity. The denominational media treatments of social sustainability show that the specific religious beliefs of each denomination can be related to the choices made among the components of the conceptual content of social sustainability. No doubt, the study of this report could highlight a religious form of media instrumentalization of social sustainability.

### 4.5. The Denominational Specificity in the Treatment of Social Sustainability

**Figure 5:**
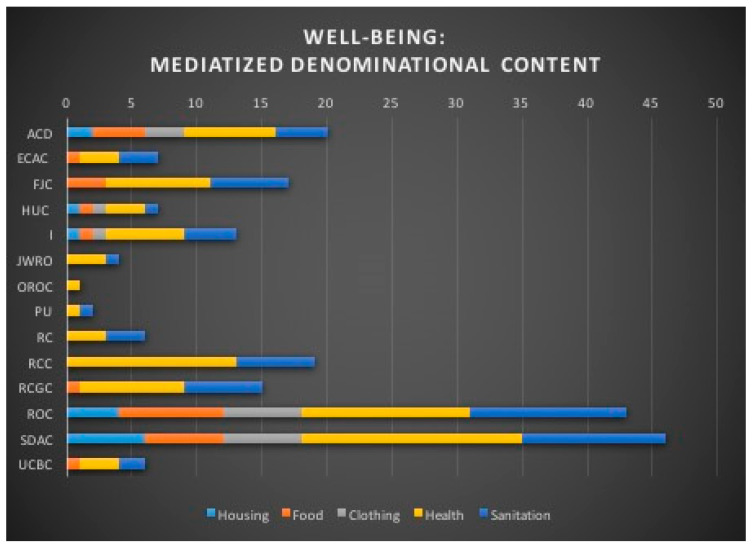
Well-being: mediatized denominational content.

The religious mediatization of the issues related to social sustainability is conditioned by the editorial policies and specificities of media production which follow the institutional and organizational injunctions of each denomination. Thus, in the denominational content of media productions, references related to the “Physical well-being” level are found to different degrees, not only in terms of quantitative accumulation, but also in terms of thematic representation (Figure 5). Thus, OROC deals with one theme, JWRO, PU, RC and RCC each deal with two themes, while ECAC, FJC, RCGC and UCBC each deal with three themes and only ACD, HUC, I, ROC and SDAC take control of all themes (Table 7). The five denominations that capture all media themes represent two different religions—Islam and Christianity—and four Christian constituents—Orthodoxy, Catholicism, Protestantism and Neo-Protestantism. This illustrates that the denominational dogmatic content is not an issue in the editorial choices.

**Figure 6:**
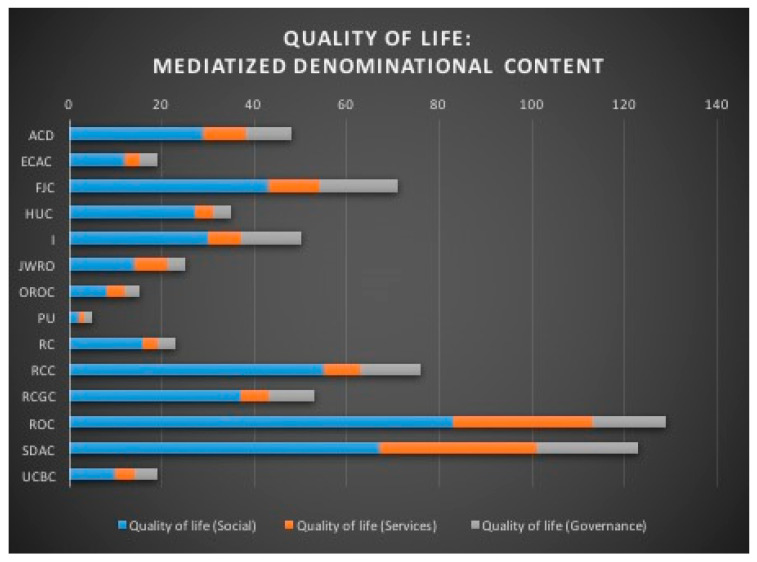
Quality of life: mediatized denominational content.

Also, in the denominational content of media products, references related to the “Quality of life” level are found to different degrees, not only in terms of quantitative accumulation, but also in terms of thematic representation (Figure 6). On the other hand, here, all denominations take up all the themes linked to the “Quality of life” (Table 8). This is not surprising in view of the work that highlights the link between religion and quality of life [141,142,143].

Considering these sets of results, it becomes clear that most denominational editorial policies do not or are not able to pursue, as a media objective, the full thematic coverage of social sustainability. This situation undoubtedly impacts the production of the denominational meaning of social sustainability, but, also promotes inter-denominationalism as an approach in favor of social sustainability.

### 4.6. The Accentuated and Concordant Intensity of the Mediatization of Certain Indicators of Social Sustainability

**Figure 7:**
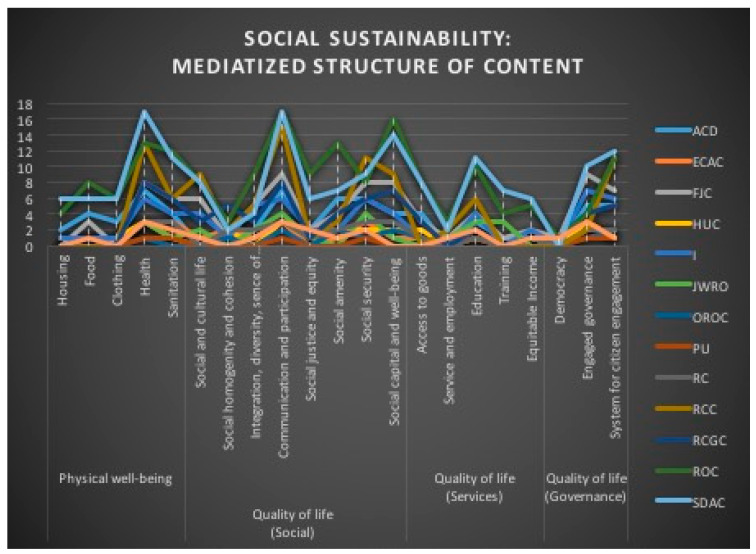
Social Sustainability: mediatized structure of the content.

The religious mediatization of the issues related to social sustainability is dominated by five ascending thematic trends in the media production of the majority of denominations—Health, Communication and Participation, Social Capital and Well-being, Education and Engaged Governance—and by four descending thematic trends for the majority of the denominations—Social Homogeneity, Service, Employment and Cohesion, and Democracy (Figure 7). The distribution of indicators by denomination, considering the total of 99 media productions, also shows that only six thematic trends are common to all denominations (Table 9). In the media coverage, the particular density of those five thematic trends highlighted by our results, density correlated with the six identified thematic trends, shows the design of a collective choice that could serve as a basis for the inter-denominational approach of social sustainability.

## 5. Discussion

The results presented above call for several observations:

Collectively, we understand these results as a contribution of the Romanian religious media—denomination by denomination—to the promotion of social sustainability in connection with the socio-political credit of complementarity and subsidiarity that the State grants to recognized religious denominations in Romania: “*Recognizing the specific contribution of religious faiths in the edification of the common good, and the particularities of the manner of involvement of religious faiths in Romania in societal life, due to their specific world views, the State Secretariat for religious affairs views as beneficial the affirmation of religious organizations in the Romanian public sphere as social actors that may contribute to the sustainable development of Romanian society*” ([125], p. 25). In this context, these results are part of a national program that can be interpreted both in the sense of valuing and instrumentalizing religion in the public sphere.

Then, contextually, the COVID-19 pandemic is not a crisis that generates, maintains or develops the religious mediatization of social sustainability for all official denominations in Romania. However, the total absence of media production on sustainability for certain denominations during this period does not necessarily mean they have no interest in social sustainability. It rather shows that a global health crisis is not necessarily the trigger for particular interest in this topic. This absence can also be an indicator of institutional resistance to the opening imposed inevitably and irreversibly by new communication technologies on closed and semi-closed organizations. This resistance is particularly interesting in the sense that, even if these institutions adapt to the logics of the (new) media, currently more open and less controllable, they continue to show a certain resistance to the authority of the global media and citizen agenda, attempting to maintain their traditional role as the most powerful provider of guidance, education, etc. against the background of the general crisis of institutions in our society [95,138].

Second, for the denominations that have produced media content, denominational distinctiveness in the religious mediatization of social sustainability appears as the expression of emancipation from their community condition, emancipation from the perspective of participation in the symbolic life of the public sphere. The gaps in media production and the differences in index coverage and thematic density cannot be explained by organizational reasons, or by a particular commitment to the cause of social sustainability. The strongest religious mediatization of social sustainability corresponds to the denominations for which the logics of the media have overtaken institutional logics, and the media in the broadest sense transform the communicative construction of their social world. More precisely, these gaps are explained by the fact that these denominations do not use their media for the *mediation* of spiritual content, but for the *mediatization* of this content, that is to say to produce both a socio-cultural transformation on the basis of mediated communication [82] with the subject of social sustainability, and to organize, present and highlight informational news linked to social sustainability by following the logics of the media [79,80].

Third, the religious mediatization of social sustainability is the expression of a pragmatic opposition between two symbolisms of power: the political symbolism and the religious symbolism. This opposition often generates conspiracy theories, because, “*conspiracy theories are quasi-religious representations, in that their contents, forms, and functions parallel those found in beliefs supported by institutionalized religions, though conspiracy theories lack certain features of organized religions*” ([139], p. 424). Nevertheless, the quantitative gap observed between the indicators of social sustainability in the analyzed media content shows that the religious meaning of social sustainability in the context of COVID-19 is not mediatized in the register of conspiracy theories, but in that of communicative action [105], which engages practical reason via rational debate, reason and argument. In this case, given the Covid-19 pandemic, the communication on social sustainability places particular emphasis on the theme of health due to the major urgency of the matter. Resonating with the general critiques of the political management of the crisis, it pays little attention to the topic of democracy. These results, and primarily the apparently incoherent gap between the prorata of indicators of the same theoretical order—“democracy” and “communication and participation”—are linked to the deeply Orthodox foundation of Christianity in Romania. The meaning of democracy in predominantly Orthodox Christian countries is different from the meaning of democracy developed in countries historically attached to Catholicism and / or Protestantism. In Romania, as in other “Orthodox” countries of the European Union, “*it is unthinkable that the nation exists without “the orthodox faith”. The maintenance of the confessional Orthodox society is understood as necessary for the survival of the nation and, (…) the secular state, the pluralistic society, the separation between civil society and the Orthodox Church are foreign and dangerous ideas*” ([143], p. 658). More precisely, social sustainability, a concept originally stemming from Western democracy, is treated by the religious media of Romania in a Byzantine register that plays between the fear of liberal and democratic society and the agreement with the rest of Europe.

Fourth, the religious mediatization is balanced between the two conceptual levels of social sustainability, showing that the pandemic has no impact on the representation of social sustainability as the capacity of the community of believers can develop processes and structures that meet current needs and, at the same time, it supports the future maintenance of the health of this national trans-denominational public. The more marked is the impact on certain internal themes at each level, —far from representing a functional lack of coordination between institutions and religious organizations, —the more it indicates an adapted responsiveness to the stimuli of media news. This is a rather positive sign for the community recognition of the effects of social sustainability. The findings reflect a media presence that appears to be out of step with the manifest commitment on the ground of each of these denominations and with the recognition of this commitment by the State. Thus, for example, contrary to the results of this study, the State Secretariat for Religious Affairs notes that “*the RCC is engaged in a wealth of charitable activities, the church has established kindergartens, orphanages, homes for the elderly, soup kitchens, and medical clinics and centers through its “Caritas” associations, other associations, and directly through parishes and monastic organizations*” ([125], p. 136) and that the PU develop a strong social activity “*organized by the denomination in hospitals, homes for the elderly, placement centers for children, and penitentiaries through its local churches and the specialized institutions*” ([125], p. 152). However, present research is not intended to quantify the investment in social sustainability denomination by denomination, but to highlight the impact in terms of the corporate media exposure of the religious institutions in the context of the Covid-19 crisis in relation to the concept of social sustainability.

Fifth, it should be noted that the religious mediatization of social sustainability to significantly different degrees, in terms of quantitative accumulation and thematic representation, produces fragmented denominational public spheres which, in a crisis situation, raise the question of the need for a complementary political strategy. At the same time, the cases of ROCs and SDACs suggest that social sustainability could play the role of an inclusive intercultural intermediary and mediator. Indeed, ROC and SDAC despite their links to different symbolism, institutional functioning and organizational visions, appear with comparable media performances, which blur identity boundaries by separating them and sometimes opposing them in the Romanian context.

A sixth observation concerns the denominational conditioning of the religious mediatization of social sustainability. While the logics of the media prevailing over the institutional logics, the denominational specificity in the treatment of social sustainability is permanently presented in the content of media products. The fact that some denominational media only deal with certain themes cannot be taken as proof of partial adherence to the concept and meaning of social sustainability. This discrepancy is rather the evidence of a kind of internal tension for each denomination, resulting from strategies of taking control of media logics. This explains why the content of media productions, considered denomination-by-denomination, covers the themes of social sustainability to significantly different degrees.

Finally, in times of crisis, subjects related to the immediately sensitive practical context can thematically dominate the religious mediatization of social sustainability. The practical context combines three dimensions: “*the technique, that is to say the how?, the method, the operability, the technology including its cultural dimension; the human kind, that is to say the who?, the individual, the anthropological grounding; the effectuation, that is to say the what?, the aim of the action, the set of actions and achievements as a productive force*” ([91], p. 213). 

During the COVID-19 pandemic, the issues of social sustainability on which the denominational media focus their information production are those relating to subjects which, beyond the collective experience, ultimately affect the personally of the believer, —through experiences reducible to the level of individual experience: personal suffering related to their own health, personal fear following their own lack of information, personal discomfort with regard to their own representations of well-being.

In contrast to this tendency, the results of our research show that, in times of crisis, the religious mediatization of social sustainability is low in discursive content on themes that culminate inevitably in the collective experience. This orientation could be explained by the fact that the lockdown conditions and social distancing inflicted by the pandemic have imposed practices and behaviors such as: (a) the control of movements and public meetings, (b) the fear of the other and the physical and social distancing (c) the authoritarian and centralized political decision-making process in an urgent manner and without debate and consultations with citizens—that are judged not being compatible with social homogeneity, cohesion and certain fundamental values of democracy.

Across the Romanian religious media landscape, the concept of social sustainability is not distorted. Social indicators are quantitatively dominant over administrative and political indicators in their internal configuration. The religious media production on the Covid-19 pandemic thus reflects popular discontent following the military injunction to suspend religious services in places of worship [144] by emphasizing the attachment of the religious institutions to the social commitment required by faith in general and, in this case in particular, by Christian faith.

## 6. Conclusions

The objective of this research was to show and explain how religious mediatization had impacted the meaning of social sustainability during the COVID-19 crisis. In relation to this objective, the analysis of the results obtained calls for four concluding remarks.

First, the inclusion of these results in the field of information and communication sciences provides a disciplinary perspective that enriches how to view human and social sciences on social sustainability. This perspective is consistent with the topicality of the human existence unprecedented percolation by the media. More precisely, this disciplinary positioning intends to explore the association of meaning between new information and communication technologies, well-being and the ability to use it as an index of quality of life.

Second, the main research question of this study and the observation features, aimed to understand the impact of the mediatization of social sustainability under the simultaneous influence of two constraints: (a) the health crisis caused by the COVID-19 pandemic and (b) the institutionalized and organized faith. The major issue was to understand and explain the shaping in the public sphere of social sustainability as a figure of social transformation.

Third, the results of this research allow us to observe multiple gaps, disparities and asymmetries in the religious media production of the meaning of social sustainability during the COVID-19 pandemic. The specificities which mark these results appear in the perspective of mediatization at three levels of meanings, which overlap and become entangled in the production of this meaning: (a) the level of the content of the very concept of social sustainability, (b) the institutional and organizational level of beliefs at work and (c) the level of media systems and logics. These three levels generate variables to be taken into account in a crisis situation.

Finally, the treatment of the results highlighted the major communication issues of social sustainability against the background of a tension that is now unavoidable between institutional and organizational logics, on the one hand, and media logics, on the other. Thus, the research has highlighted the pragmatic need to consider the conceptual flexibility of the meaning of social sustainability in crisis contexts, and the major role of religious mediatization in the process of actualizing this meaning in the public sphere.

## Figures and Tables

**Table 1:** Recognized denominations in Romania.

Recognized Denominations	Acronym
Armenian Church Diocese	ACD
Christian Church of the Gospel in Romania	CCG
Evangelical Church of the Augsburg Confession of Romania	ECAC
Evangelical Lutheran Church of Romania	ELC
Federation of Jewish Communities in Romania—Jewish Faith	FJC
Hungarian Unitarian Church	HUC
Islam	I
Jehovah’s Witnesses Religious Organization	JWRO
Old-Rite Orthodox Church of Romania	OROC
Pentecostal Union—The Apostolic Church of God of Romania	PU
Reformed Church of Romania	RC
Roman Catholic Church	RCC
Romanian Church United with Rome, Greek Catholic	RCGC
Romanian Evangelical Church	REC
Romanian Orthodox Church	ROC
Serbian orthodox Diocese	SOD
Seventh-day Adventist Church in Romania	SDAC
Union of Christian Baptist Churches in Romania	UCBC

**Table 2:** Indicators of Social Sustainability (source: Hajirasouli and Kumarasuriyar, 2016) [137].

Indicators of Social Sustainability		
Physical well-being	Housing	
	Food	
	Clothing	
	Health	
	Sanitation	
Quality of life/equity	Social	Social and cultural life
		Social homogeneity and cohesion
		Integration, diversity, sense of place
		Communication and participation
		Social Justice and equity
		Social amenity
		Social security
		Social capital and well-being
	Services	Access to goods
		Service and employment
		Education
		Training
		Equitable Income
	Governance	Democracy
		Engaged governance
		System for citizen engagement

**Table 3:** Denominational media production of social sustainability (average density of indicators).

Denominations	Number of Media Products	Indicator Media Coverage	Average Density/ Media Product
ACD	7	68	16.71%
ECAC	3	26	8.66%
FJC	9	88	9.77%
HUC	3	27	9%
I	7	63	9%
JWRO	4	29	7.25%
OROC	1	16	16%
PU	1	7	7%
RC	3	29	9.66%
RCC	15	95	6.33%
RCGC	8	68	8.5%
ROC	17	172	10.11%
SDAC	18	169	9.38%
UCBC	3	25	8.33

**Table 4:** Social sustainability indicators (average density of indicators).

Indicators		%
Physical well-being	Housing	1.71%
	Food	3.07%
	Clothing	2.08%
	Health	9.95%
	Sanitation	6.63%
Quality of life (Social)	Social and cultural life	4.79%
	Social homogeneity and cohesion	2.21%
	Integration, diversity, sense of place	4.42%
	Communication and participation	11.05%
	Social justice and equity	3.31%
	Social amenity	5.28%
	Social security	7.86%
	Social capital and well-being	7.86%
Quality of life (Services)	Access to goods	3.56%
	Service and employment	1.22%
	Education	6.14%
	Training	1.96%
	Equitable income	2.45%
Quality of life (Governance)	Democracy	0.36%
	Engaged governance	6.38%
	System for citizen engagement	7.61%

**Table 5:** Conceptual levels of social sustainability (average density of indicators).

Conceptual Levels of Social Sustainability	%
Physical well-being (Housing)	3.63%
Physical well-being (Food)	6.48%
Physical well-being (Clothing)	4.41%
Physical well-being (Health)	21.01%
Physical well-being (Sanitation)	14.02%
Quality of life (Social)	30.85%
Quality of life (Services)	10.12%
Quality of life (Governance)	9.48%

**Table 6:** Conceptual levels of social sustainability by denomination (average density of indicators).

Conceptual Levels of Social Sustainability		ACD	ECAC	FJC	HUC	I	JWRO	OROC	PU	RC	RCC	RCGC	ROC	SDAC	UCBC
Physical well-being	Housing	2	0	0	1	1	0	0	0	0	0	0	4	6	0
	Food	4	1	3	1	1	0	0	0	0	0	1	8	6	1
	Clothing	3	0	0	1	1	0	0	0	0	0	0	6	6	0
	Health	7	3	8	3	6	3	1	1	3	13	8	13	17	3
	Sanitation	4	3	6	1	4	1	0	1	3	6	6	12	11	2
Quality of life	Social	4	2	5	3	4	2	1	0	2	7	5	10	8	1
	Services	2	1	2	1	1	1	1	0	1	2	1	6	7	1
	Governance	3	1	6	1	4	1	1	1	1	4	3	5	7	2

**Table 7:** Mediatized denominational content (average density of indicators).

Denominational Content		ACD	ECAC	FJC	HUC	I	JWRO	OROC	PU	RC	RCC	RCGC	ROC	SDAC	UCBC
WELL-BEING	Housing	2	0	0	1	1	0	0	0	0	0	0	4	6	0
	Food	4	1	3	1	1	0	0	0	0	0	1	8	6	1
	Clothing	3	0	0	1	1	0	0	0	0	0	0	6	6	0
	Health	7	3	8	3	6	3	1	1	3	13	8	13	17	3
	Sanitation	4	3	6	1	4	1	0	1	3	6	6	12	11	2

**Table 8:** Denominational content (average density of indicators).

Denominational Content		ACD	ECAC	FJC	HUC	I	JWRO	OROC	PU	RC	RCC	RCGC	ROC	SDAC	UCBC
Quality of Life	Social	29	12	43	27	30	14	8	2	16	55	37	83	67	10
	Services	9	3	11	4	7	7	4	1	3	8	6	30	34	4
	Governance	10	4	17	4	13	4	3	2	4	13	10	16	22	5

**Table 9:** Indicators (media average).

INDICATORS		ACD	ECAC	FJC	HUC	I	JWRO	OROC	PU	RC	RCC	RCGC	ROC	SDAC	UCBC
Physical well-being	Housing	2	0	0	1	1	0	0	0	0	0	0	4	6	0
	Food	4	1	3	1	1	0	0	0	0	0	1	8	6	1
	Clothing	3	0	0	1	1	0	0	0	0	0	0	6	6	0
	Health	7	3	8	3	6	3	1	1	3	13	8	13	17	3
	Sanitation	4	3	6	1	4	1	0	1	3	6	6	12	11	2
Quality of life (Social)	Social and cultural life	0	1	6	0	4	2	0	0	0	9	3	8	8	1
	Social homogeneity and cohesion	2	2	1	2	1	0	1	0	2	2	5	3	2	0
	Integration, diversity, sense of place	2	1	5	0	5	2	0	0	2	5	3	9	4	1
	Communication and participation	7	3	9	3	6	4	2	1	3	15	8	17	17	3
	Social justice and equity	2	0	2	1	2	1	0	0	1	1	1	9	6	2
	Social amenity	6	1	4	2	2	0	2	0	2	3	4	13	7	1
	Social security	6	3	8	2	6	4	1	1	3	11	6	8	9	2
	Social capital and well-being	4	1	8	2	4	1	2	0	3	9	7	16	14	0
Quality of life (Services)	Access to goods	4	1	3	2	1	0	1	0	1	0	3	8	8	0
	Service and employment	0	0	1	0	0	1	0	0	0	2	0	3	2	1
	Education	3	2	4	1	4	3	2	1	1	6	2	10	11	2
	Training	1	0	1	0	0	3	0	0	0	0	0	4	7	0
	Equitable income	1	0	2	1	2	0	1	0	1	0	1	5	6	1
Quality of life (Governance)	Democracy	0	0	1	0	0	0	0	0	0	0	1	1	0	1
	Engaged governance	5	3	9	3	7	2	1	1	2	2	4	4	10	3
	System for citizen engagement	5	1	7	1	6	2	2	1	2	11	5	11	12	1
